# [Retracted] Imatinib inhibits oxidative stress response in spinal cord injury rats by activating Nrf2/HO‑1 signaling pathway

**DOI:** 10.3892/etm.2023.12280

**Published:** 2023-10-30

**Authors:** Limin Liu, Jingyuan Zhou, Yufeng Wang, Tengmin Qi, Zengshun Wang, Linxu Chen, Nananxiu Suo

Exp Ther Med 19:597–602, 2020; DOI: 10.3892/etm.2019.8270

Following the publication of this paper, the authors personally requested that the article be retracted on account of their subsequent realization that they had used improper experimental methods, combined with general concerns about the published data and the fact that a large number of the experimental findings were found to be irreproducible. Independently of the authors’ request, it was drawn to the Editor’s attention by a concerned reader that the immunohistochemical data shown in the various data panels in Fig. 1 on p. 599 were overlapping, such that data which had been included in the figure to represent different experimental conditions may have been derived from the same original source(s). In addition, control western blotting data shown in Fig. 3 on p. 600 were strikingly similar to data appearing in different form in other articles written by different authors at different research institutes, which had either already been published or were under consideration for publication at around the same time.

Owing to the fact that contentious data in the above article had already been published prior to its submission to *Experimental and Therapeutic Medicine,* and also based on an overall lack of confidence in the presented data, the Editor has accepted the request of the authors to retract this paper from the Journal. The Editor apologizes to the readership for any inconvenience caused.

